# Valve-Sparing Root Replacement: Reimplantation Technique

**DOI:** 10.1016/j.cjco.2023.11.022

**Published:** 2023-12-04

**Authors:** Yuan Qiu, Munir Boodhwani

**Affiliations:** a Division of Cardiac Surgery, University of Ottawa Heart Institute, Ottawa, Ontario, Canada

**Valve-sparing root replacement (VSRR) has been shown to be an effective treatment for aortic root pathology with or without aortic insufficiency (AI) that preserves the patient’s native aortic valve.**^1^, ^2^, ^3^, ^4^**The 2 VSRR techniques—remodelling (first described by Dr Yacoub in the 1980s) and reimplantation (first described by Dr David in the 1990s)**—**have evolved and have come to share more similarities over time.****^4^** **In this paper, we describe the nuances of VSRR using the reimplantation technique, along with special perioperative considerations.**

## Preoperative Assessment

Aortic diameter should be measured at various points, typically using computed tomography. This measurement determines the extent of distal aortic repair needed, which can impact how the root operation is conducted. Computed tomography also enables assessment of coronary anatomy and leaflet calcification. The degree and mechanisms of AI, as well as leaflet anatomy and motion, should be assessed using transthoracic echocardiography, with transesophageal echocardiography (TEE) performed preoperatively as needed to enhance image quality. Alternative valve choices should be discussed with the patient in case a repair is deemed to be infeasible.

## Operative Technique

### Aortotomy and exposure

Cardiopulmonary bypass typically is instituted with distal aortic and right atrial cannulation. Antegrade ± retrograde cardioplegia is used, depending on the degree of AI. Following aortic clamping, a transverse aortotomy is performed about 1 cm above the sinotubular junction (STJ), keeping the STJ intact. Polypropylene commissural retraction sutures are placed at the level of the commissures, and the distal aorta is retracted cephalad.^2^ The aortic valve and root then are inspected thoroughly. Although this article focuses on repair of a trileaflet aortic valve, repair of a bicuspid aortic valve (BAV) using similar techniques has been shown to have similar outcomes over a 10-year follow-up period.^3^

### Valve assessment and leaflet repair

**Figure 1:**
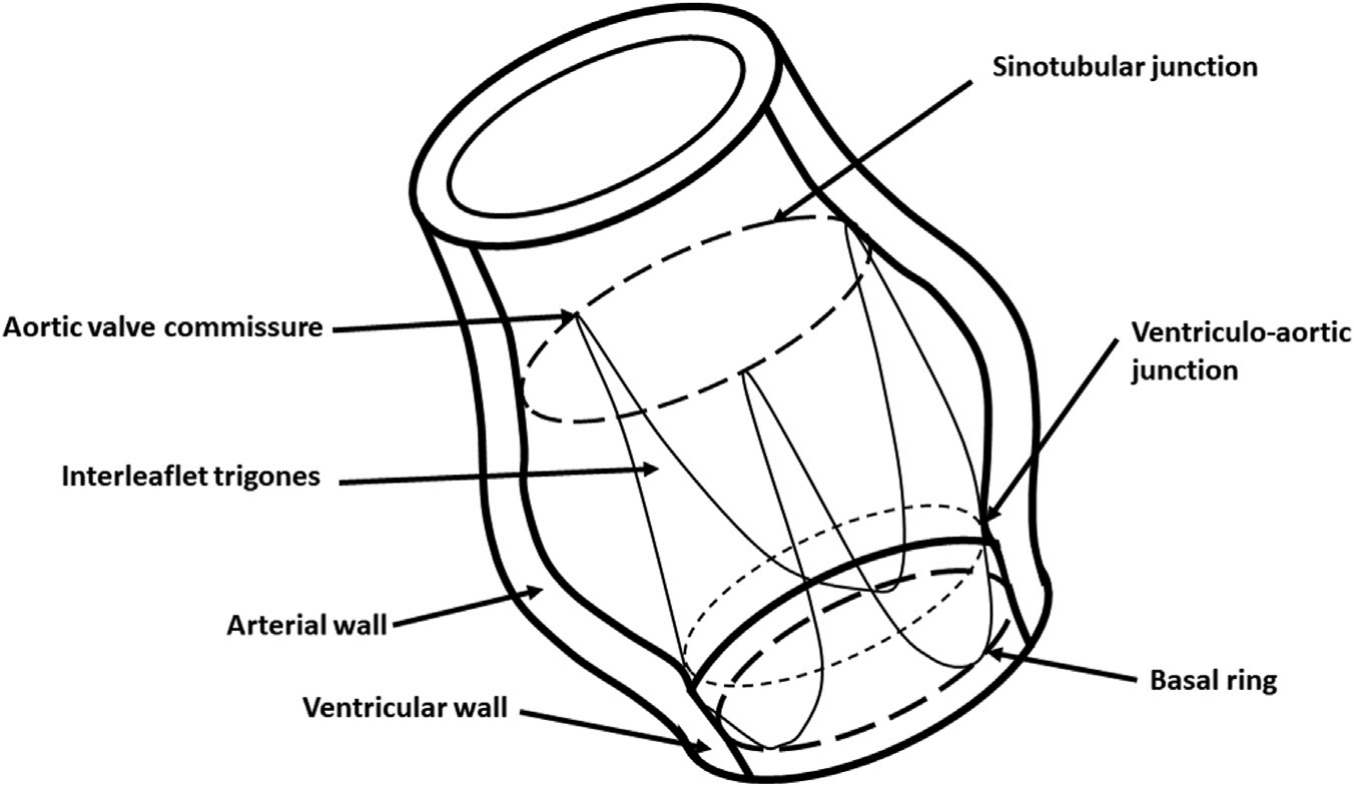
Aortic root anatomy. Adapted from Kırali et al.^7^ under Creative Commons Attribution 3.0 Unported (CC BY 3.0 DEED) license (https://creativecommons.org/licenses/by/3.0/).

An understanding of the patho-anatomy of the aortic valve and root is crucial for many aspects of this procedure (Fig. 1). The functional aortic annulus (FAA) includes the STJ and the ventriculo-aortic junction (VAJ), which together provide the support structure for the valve cusps. Alterations in the cusps (prolapse, restriction, calcification, fenestrations) or in the FAA (STJ dilation, VAJ dilation, or both) can contribute to AI.^1^ Often, multiple mechanisms contribute to AI, and both the FAA and cusps need to be restored to normal (or functional) geometry for a durable repair.

The leaflets, sinuses, and annulus should be inspected, examining for leaflet tissue quality, mobility, and evidence of cusp prolapse. The main reasons a valve would be deemed unrepairable are the presence of significant calcification, large and multiple fenestrations (particularly if they impact coaptation or are ruptured), and significant leaflet restriction if the geometric height is inadequate (< 17 mm for trileaflet valves, and < 20 mm for a nonfused cusp of a BAV).^5^ Any leaflet repair that is needed to confirm the preservability and repairability of the valve should be performed at this stage (eg, leaflet shaving, decalcification, BAV raphe release, management of fenestrations), before committing to the VSRR procedure. However, cusp prolapse repair should be performed after the root procedure has been completed, as changes in annular geometry can affect the degree of cusp repair needed.

### Aortic root preparation

The aortic root is dissected externally down to the level of the VAJ to enable complete access to the FAA, except at the right/non commissure where this dissection is limited by the presence of the membranous septum. This dissection also enables circumferential VAJ support and reduction by securing the graft at this level. The noncoronary sinus should be dissected first, down to the level of leaflet insertion, and the sinus of Valsalva resected, leaving about 5-7 mm of aortic tissue.^2^ The right coronary and then the left coronary buttons are harvested. External dissection is limited by the membranous septum and ventricular muscle (Video 1 , view video online).

At the right/left commissure, the pulmonary artery and the right ventricle are detached from the aortic root. Dissection is performed through the aorto-pulmonary ligament, a white fibrous tissue, then through the yellow-appearing fat tissue underneath, and then into the muscle. This deep-tissue dissection enables a circumferential and robust annuloplasty that would be difficult to do with the coronary arteries intact. Ostial cardioplegia catheters are secured in the coronary buttons for intermittent delivery of cardioplegia that can be performed at appropriate time intervals without interrupting the flow of the operation.

### Sizing

An appropriately selected graft size is a crucial step in the VSRR to restore optimal root, annular, and leaflet configuration. We use a graft-sizing technique based on the principle that the height of the commissure remains relatively constant in the setting of aortic root and FAA dilation, and, in a normally functioning aortic valve, the height of the commissure is equal to the external diameter of the STJ.^6^

**Figure 2:**
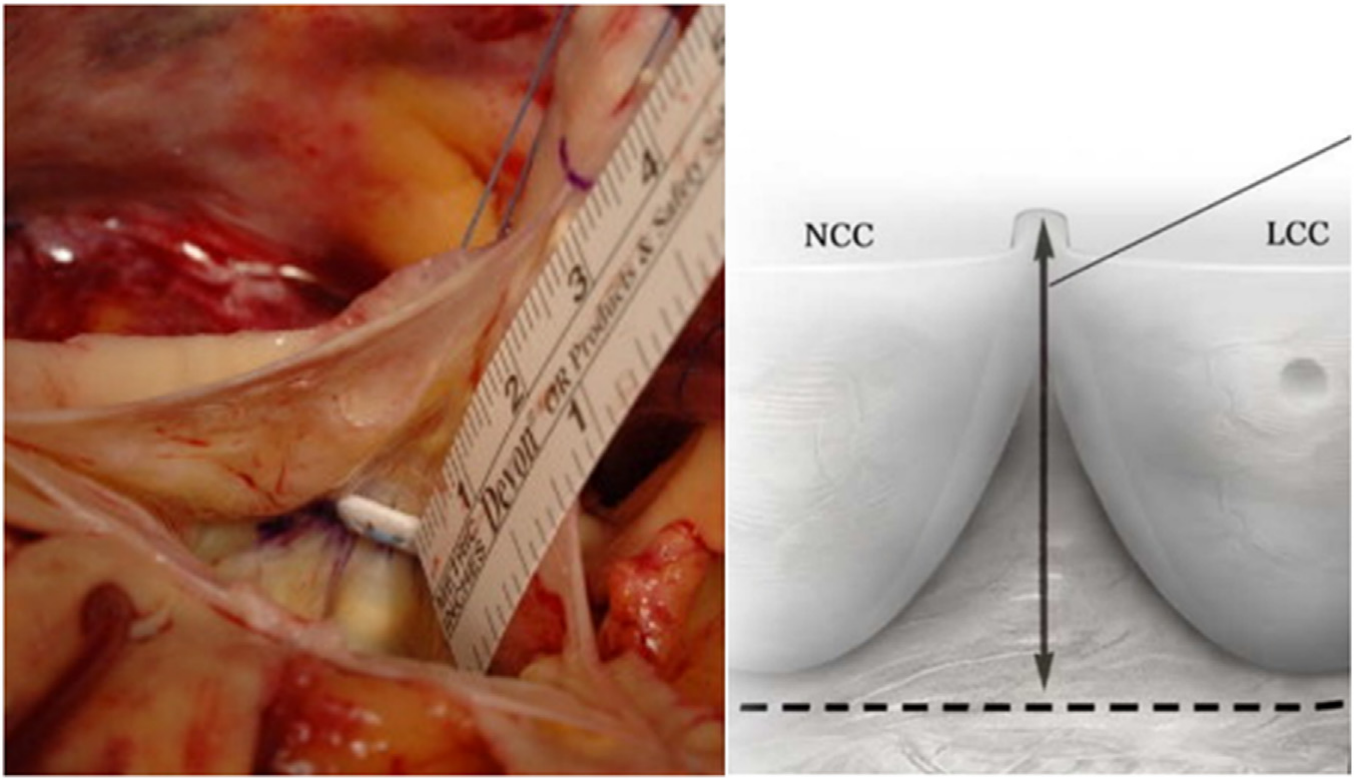
Measurement of the height of the commissure. LCC, left-coronary commissure; NCC, noncoronary commissure. Adapted from de Kerchove et al.^6^ with permission from Elsevier.

The height of the commissure at the non/left commissure is measured by drawing a line between the nadirs of the 2 cusps, which mark the base of the interleaflet triangle, and then measuring from this line to the top of the commissure (Fig. 2). This measurement will be the same as the size of the graft chosen. For example, a 28-mm distance correlates to a 28-mm graft. If the measurement does not correspond to a labeled graft size, the next larger size graft should be used.^6^ This sizing technique allows for restoration of normal aortic valve geometry and function and can be used with a straight graft and those with a preformed sinus of Valsalva segment. We prefer to use a graft with preformed sinuses of Valsalva.

The graft size is an important determinant of the final annular diameter of the STJ and VAJ. If undersized, the excessive cusp tissue will result in cusp prolapse, low coaptation, and AI. If the graft is too large, this can result in the valve cusps stretching outward, cusp restriction, and a central coaptation defect.

### Proximal suture line

Pledgetted 2-0 braided sutures are passed from inside to outside of the aorta with the pledgets on the inside, starting from the non/left commissure and moving clockwise. The proximal suture line along the fibrous portion of the aortic annulus is sewn at the base of the interleaflet triangle, except at the right/non commissure where external dissection is limited by the membranous septum. These sutures should be placed along the lowest portion of the freely dissected aortic root. The proximal suture line will be slightly higher at the right/non commissure but will be uniplanar everywhere else. This serves as the annuloplasty of the VAJ.

The graft is prepared by removing the cuff, and a small indentation is made at the right/non commissure. The size of this indentation is determined by subtracting the height from the proximal suture line to the top of the commissure at the right/non commissure from the total commissural height. Sutures are then passed through the base of the graft and the graft is tied into place around the native aortic root (Video 2 , view video online).

### Second/reimplantation suture line

The commissures are first reimplanted using 4-0 polypropylene sutures while the prosthesis and the native commissures are pulled up and then tied into place. Radial traction is applied on 2 adjacent commissural sutures, which reveals the “line of implantation.” The suture line is run in small steps, passing the suture from outside the prosthesis to inside and through the aortic wall, staying close to the annulus, following the crown shape of the aortic annulus (Video 3 , view video online). When a total arch replacement is being performed concomitantly, the graft can be transected 1 cm above the new STJ, to enable better visualization and facilitate easier completion of this suture line.

### Valve reassessment

After valve reimplantation, the leaflets are re-examined for any unmasked prolapse, symmetry, and the height and depth of coaptation. Cusp prolapse can be repaired using a variety of techniques, including leaflet resection and primary repair, free margin resuspension, and central free margin plication, which is used most frequently. Cardioplegia is administered through the distal end of the graft, with partial clamping to distend the new aortic root and to assess root pressure and signs of left ventricular dilation. Intraoperative TEE is performed at this time to assess valve competency. The cardioplegia solution is suctioned slowly out of the prosthesis, without distorting the leaflets. This step provides another opportunity for visual assessment of the aortic valve in its physiologically closed state, as well as of the height and area of coaptation.

### Coronary button reimplantation and distal anastomosis

The ophthalmic cautery is used to make holes, keeping in mind the native position of the coronary arteries, and 5-0 prolene sutures are used to anastomose the coronary buttons. The dilated portions of the aorta are resected, and the distal anastomosis is performed at the level of the normal-caliber ascending aorta, with a running 4-0 prolene suture (Video 4 , view video online). The heart is de-aired, and the cross-clamp is removed from the aorta.

Post–cardiopulmonary bypass TEE should show no residual AI and excellent leaflet coaptation. The mean gradient and left ventricular outflow tract gradient should be measured. The aortic valve area also can be assessed. Predictors of repair failure on postrepair echocardiographic assessment include inadequate coaptation length (< 5 mm), ineffective height (< 9 mm), residual AI—particularly if eccentric—and a large residual annulus (diameter > 26 mm).

## Discussion

The VSRR with the reimplantation technique enables remodelling of the aortic annulus and can accommodate different geometries. The VSRR provides both internal and external support for both components of the aortic annulus, the STJ, and the VAJ. We recommend echocardiographic follow-up within the first 3 months postrepair, and annually thereafter. Overall, this technique is robust and reproducible, allows for preservation of aortic valves, and results in consistent and excellent long-term outcomes.

## Supplementary Material

Video 1 Aortic root preparation.

Video 2 Proximal suture line and graft preparation.

Video 3 Reimplantation suture line.

Video 4 Coronary button reimplantation and distal anastomosis.
